# Octopaminergic modulation of a fly visual motion-sensitive neuron during stimulation with naturalistic optic flow

**DOI:** 10.3389/fnbeh.2013.00155

**Published:** 2013-10-29

**Authors:** Diana Rien, Roland Kern, Rafael Kurtz

**Affiliations:** ^1^Department of Neurobiology, Faculty of Biology, Bielefeld University, Bielefeld, Germany; ^2^Department of Neurobiology and Centre of Excellence “Cognitive Interaction Technology”, Faculty of Biology, Bielefeld University, Bielefeld, Germany

**Keywords:** neuromodulation, octopamine, state dependence, visual motion processing, invertebrate, natural stimuli, optic flow

## Abstract

In a variety of species locomotor activity, like walking or flying, has been demonstrated to alter visual information processing. The neuromodulator octopamine was shown to change the response characteristics of optic flow processing neurons in the fly's visual system in a similar way as locomotor activity. This modulation resulted in enhanced neuronal responses, in particular during sustained stimulation with high temporal frequencies, and in shorter latencies of responses to abrupt onsets of pattern motion. These state-dependent changes were interpreted to adjust neuronal tuning to the range of high velocities encountered during locomotion. Here we assess the significance of these changes for the processing of optic flow as experienced during flight. Naturalistic image sequences were reconstructed based on measurements of the head position and gaze direction of *Calliphora vicina* flying in an arena. We recorded the responses of the V1 neuron during presentation of these image sequences on a panoramic stimulus device (“FliMax”). Consistent with previous accounts, we found that spontaneous as well as stimulus-induced spike rates were increased by an octopamine agonist and decreased by an antagonist. Moreover, a small but consistent decrease in response latency upon octopaminergic activation was present, which might support fast responses to optic flow cues and limit instabilities during closed-loop optomotor regulation. However, apart from these effects the similarities between the dynamic response properties in the different pharmacologically induced states were surprisingly high, indicating that the processing of naturalistic optic flow is not fundamentally altered by octopaminergic modulation.

## Introduction

How animals perceive their environment depends on their behavioral state. There is growing evidence from various animal models that locomotor activity like walking or flying modulates the processing of sensory information (Rind et al., 2008; Chiappe et al., 2010; Niell and Stryker, 2010; Rosner et al., 2010; Andermann et al., 2011; Maimon, 2011; McArthur and Dickman, 2011; Keller et al., 2012). The neuromodulator octopamine, which is the invertebrate analogue of norepinephrine in the vertebrate neural system (Hurley et al., 2004; Sara and Bouret, 2012), is a key candidate for the control of state-dependent sensory processing (Roeder, 2005). In locusts, octopamine is released in high quantities during flight (Goosey and Candy, 1980). Mutant flies lacking the enzyme for the synthesis of octopamine from its precursor tyramine are impaired in flight initiation and maintenance (Brembs et al., 2007). In flies as well as in locusts, administration of octopamine or its agonist chlordimeform (CDM) was shown to affect visual processing (Bacon et al., 1995; Longden and Krapp, 2009, 2010; Jung et al., 2011; Haan et al., 2012; Rien et al., 2012).

Locomotor activity was shown to alter the response properties of visual motion-processing interneurons, the lobula plate tangential cells (LPTCs). LPTCs are located in the posterior part of the fly's third visual neuropile, and most of them can be individually identified based on their unique properties. LPTCs perform a crucial function in optic flow processing, i.e., the rapid extraction of relevant information from global visual input during locomotion (Egelhaaf et al., 2002; Borst et al., 2010). Visual motion responses of LPTCs were shown to be higher during tethered flight (Maimon et al., 2010) or walking (Chiappe et al., 2010) than during periods of rest, in particular at high temporal frequencies of grating motion (Chiappe et al., 2010; Jung et al., 2011). The state-dependent difference in dynamic response properties could be reproduced by application of CDM in restrained flies (Longden and Krapp, 2010; Jung et al., 2011). Recently, genetic methods were used in *Drosophila* to show directly that the activity of a set of octopaminergic neurons is necessary and sufficient for the state-dependent modulation of one class of LPTCs (Suver et al., 2012).

During flight in a structured environment a fly is confronted with complex optic flow, determined by its flight movements, its gaze shifts, and the structure of its environment. How the processing of natural optic flow is affected by state-dependent modulation is still unresolved, because in all previous experiments addressing state dependence periodic gratings drifting with experimenter-designed velocity profiles were used. As a first step to resolve the state dependence of natural optic flow processing we recorded an identified LPTC, the V1 neuron, during presentation of image sequences that were reconstructed from the gaze direction of a blowfly during semi-free flight in an arena (Kern et al., 2005; van Hateren et al., 2005). During panoramic replay of these image sequences we tested the effects of CDM and of the octopamine receptor antagonist epinastine (Roeder et al., 1998) on the neuronal responses.

## Materials and methods

### Electrophysiology

Female blowflies (*Calliphora vicina*, 3–6 days old) were taken from our laboratory stock and dissected according to the standards in our laboratory. The flies were briefly anesthetized with CO_2_ and fixed to a small glass plate at the dorsal thorax. The legs were removed and the wounds were sealed with bee's wax. The wings and abdomen were immobilized with bee's wax. The proboscis was stretched out and fixed to the thorax. The head was opened from behind and the air sacs were removed. Ringer's solution (for composition see Kurtz et al., 2001) was used to prevent desiccation of the brain and to fill a glass pipette that served as indifferent electrode. The orientation of the fly's head was aligned according to the deep pseudopupil in the frontal region of both eyes (Franceschini, 1975).

The activity of the V1 neuron was recorded extracellularly in its output arborization in the right brain hemisphere. Borosilicate glass electrodes (GC150TF-10, Clark Electromedical, Edenbridge, UK) with an outer diameter of 1.5 mm were pulled using a Brown-Flaming electrode puller (P97, Sutter Instruments, San Rafael, CA, USA) and filled with 1 M KCl which resulted in an electrode resistance of 1–5 MΩ. The V1 neuron is unambiguously identifiable by its sensitivity to downward motion in the frontal to frontolateral part of the visual field contralateral to the recording site (Hausen, 1976). Data were only collected from neurons that displayed sufficiently large spike amplitudes relative to the background noise level. The raw signals were sampled at 20 kHz (DT 3001; Data Translation, Marlboro, MA, USA) and stored via the MATLAB data acquisition toolbox (The MathWorks Inc., Natick, MA, USA) for offline analysis.

### Visual stimulation

The trajectory of a semi-free flight was kindly provided by Dr. J. H. van Hateren (University of Groningen, Groningen, The Netherlands). The data were obtained by monitoring the voltage induced in miniature sensor coils mounted on the heads of blowflies flying in a cubic arena (edge length 0.4 m), placed in a Helmholtz coil (Schilstra and van Hateren, 1998). The walls of the arena were covered with photographs of herbage (Schilstra and Hateren, 1999). From the known gaze direction and interior of the box the visual input could be reconstructed and presented on a panoramic LED display, called FliMax (Lindemann et al., 2003). We use the terms “self motion” and “self-motion components” in the following text to refer to the locomotion of the fly in the original semi-free flight experiments. It is important to note that these terms are used for simplicity to characterize the corresponding optic flow, although all our neuronal recording experiments were performed in immobilized flies. The FliMax has an icosahedral shape, covering more than 200° in azimuth and 150° in elevation of the fly's visual field with a spatial resolution of 2.3°. In the present version of the FliMax 7168 ultrabright LEDs, which are arranged in rhomboids, can display 190 levels of light intensities (Liang et al., 2011). The stimulus was displayed at a presentation rate of 354 frames per second. To avoid transient on- and offset responses each stimulus sequence commenced with an intensity ramp of 0.5 s duration bringing each LED from 50% intensity to the value of the first stimulus image and vice versa. An interval of at least 5 s elapsed between consecutive stimulus presentations.

### Pharmacology

We applied the tissue-permeable octopamine receptor agonist chlordimeform-hydrochlorid (CDM; Sigma-Aldrich, Dorset, UK; Evans and Gee, 1980; Hollingworth and Murdock, 1980) to imitate in immobilized flies a neural sensitivity state as observed during locomotion (Longden and Krapp, 2009, 2010; Jung et al., 2011; Rien et al., 2012). A 10 μl drop of 2.6 μM CDM was directly administered to the hemolymph of the fly's head capsule (see Rien et al., 2012 for a discussion on why this concentration was chosen).

To block the effects of octopamine we used the highly specific octopamine receptor antagonist epinastine. Epinastine has a high affinity for insect neuronal octopamine receptors, whereas for other insect biogenic amine receptors like serotonin or tyramine receptors epinastine displays a 4 order of magnitude lower affinity (Roeder et al., 1998), making it an ideal candidate to scrutinize the octopaminergic modulation of signal processing. Epinastine was solved in water as a 0.1 M stock solution and stored at −18 °C. Prior to each experiment, epinastine was diluted in Ringer's solution to obtain a final concentration of 2 mM. A drop of 10 μl epinastine solution was applied to the fly's head capsule. In a series of pre-tests we found the concentration of epinastine induced by this procedure to be physiologically effective. A decrease in the resting activity of the V1 neuron was reliably produced 15–20 min after application of epinastine, and lasted until the end of the recording experiments (up to 50 min).

In locusts, injection of epinastine in high concentration (10 μl of 0.1 or 0.25 M) has been shown to lead to an immediate decrease in activity of a collision-sensitive visual neuron, the “descending contralateral movement detector” (DCMD) whereas injection of a lower concentration (0.02 M) had a smaller, delayed effect (Roeder et al., 1998). In our experiments, a concentration of 2 mM was sufficient to cause a drop in resting activity approximately 20 min after application. Higher concentrations led to a rapid complete rundown of neural activity, suggesting that these concentrations were lethal.

### Data analysis

Data were evaluated offline using custom analysis routines written in MATLAB 2011b (The MathWorks, Natick, MA, USA). The number of neurons is denoted by “N.” Visual stimulus presentation was repeated 15 times (range 12–18) for each fly and each treatment. Data were first averaged for each fly and then, if required, over the entire sample of flies. Results are given in the text as median +/− median absolute deviation, unless otherwise stated. We applied the Wilcoxon signed rank test to test for statistically significant differences between the differently treated conditions, considering a significance level of *p* < 5%.

Spikes were detected offline by thresholding the recorded potentials. The resting activity was determined in a 150 ms time window prior to motion stimulation, with the panoramic stimulus device uniformly set to mean luminance. For presentation of average peri-stimulus time-histograms spike trains were binned to a temporal resolution of 4 kHz. As a measure of the strength of octopaminergic modulation we took the difference between the responses after CDM application and the responses following subsequent epinastine application. For the analysis shown in Figures 3, 8D this measure was normalized by dividing the difference by the sum.

To quantify the similarity and the temporal relationship between the responses before and after drug application, we calculated cross-correlations between them. The cross-correlations were calculated from averaged response traces of each individual fly. We compared the maximum cross-correlation values between the responses obtained in different conditions with those of the responses obtained within a condition. The cross-correlations within a condition were obtained by splitting the data set for this condition into two halves. Due to the variability of neuronal responses and the limited size of the data set, the peak values of these cross-correlations stay below “1.” Note however, that these values should not be regarded as upper bounds, which can maximally be reached by the cross-correlations between conditions. The reason for this is the fact that the data sets for the cross-correlations within a condition are only half the size of those for the cross-correlations between conditions. We therefore also show cross-correlation values between conditions that were obtained after splitting the data sets into halves. Data sets were split by taking every second data trace in the order of recording. This produces two data sets for each condition and, correspondingly, four cross-correlation values, which were then averaged to a single value. Additionally, we analyzed the similarity of the responses within and across the different pharmacological conditions by a coherence analysis (see following section for details), which allows working with entire data sets.

We employed the coherence analysis to determine how the dynamic response characteristic of V1 is influenced by octopaminergic activity. To quantify how well the neuronal responses before and after drug application can be transformed linearly into each other, we calculated the coherence function as follows: γ^2^(*f*) = |*P*_*xy*_(*f*)|^2^/ [*P*_*xx*_(*f*)*P*_*yy*_(*f*)], where *P*_*xy*_ is the cross-spectral density of the responses before and after drug application, *P*_*xx*_ and *P*_*yy*_ are the power spectral densities before and after drug application, respectively. Coherence spectra were calculated by periodigram averaging of 50% overlapping data segments.

In a perfectly linear and noise free system the coherence γ^2^ would equal “1” at all frequencies. To estimate the noise inherent in the system, we determined the expected coherence (see Figure 7). The expected coherence is denoted as the average coherence between individual response traces and the corresponding averaged response. An expected coherence below “1” indicates system noise and therefore provides an upper bound for our estimation how the response dynamics of V1 are influenced by CDM and epinastine in a non-linear manner.

Coherence analysis was also used to analyze the relationship between particular components of the fly's self-motion and the neuronal response. For this calculation the neuronal response data was binned to a temporal resolution of 4 kHz. To obtain the same frequency for the self-motion components, their time courses were up-sampled from their original temporal resolution of 2.22 kHz by using a cubic spline interpolation. In a previous study the coherences for the translational self-motion components were found to be higher when restricting the analysis to intersaccadic intervals (Karmeier et al., 2006). We therefore followed this routine for calculating the coherences for translational self-motion components, basically using the procedures described in this study for the selection of intersaccadic intervals and for the coherence analysis.

## Results

**Figure 1:**
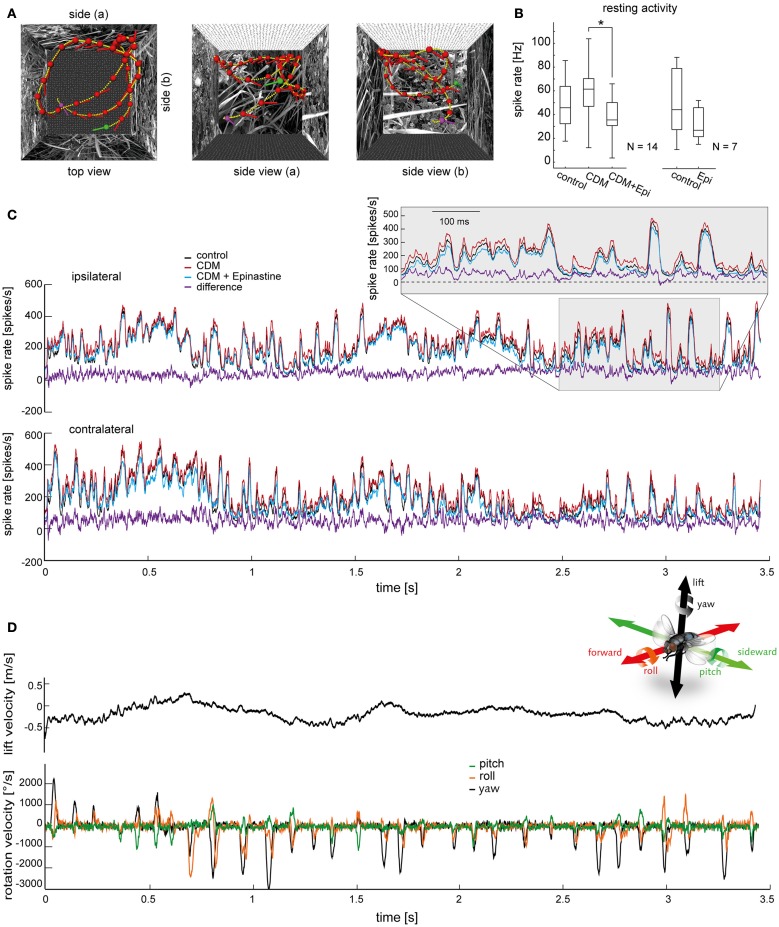
**Effects of chlordimeform (CDM) and epinastine (epi) on V1's resting activity and on the responses to naturalistic optic flow. (A)** Trajectory of a blowfly during semi-free flight in a cubic box with 40 cm edge length as seen from above (left) and from two sides. Head position and orientation are shown every 100 ms (red dots and lines; green and purple dots mark starting and end point, respectively). Small yellow dots indicate the head position every 10 ms. **(B)** Resting activity averaged over a 150-ms time window before motion onset during mean luminance. The boxplot illustrates the median as a central line and the upper/lower quartiles containing 50% of all data as box edges. The whiskers span the entire data range. *N* denotes the number of cells. The asterisk indicates statistical significance at the 5% significance level (Wilcoxon signed rank test). **(C)** Averaged responses of all V1 neurons to the stimulus illustrated in **(A)** (top, ipsilateral V1; bottom, contralateral V1). The response of the ipsilateral V1 neuron was measured directly. The responses of the same neuron to a mirror inverted version of the stimulus provided an approximation of the responses of the contralateral V1. The responses before drug application are shown in black, after CDM application in red, and after subsequent epinastine application in blue. The purple trace shows the difference between the state after CDM administration and the state after subsequent epinastine administration, which is, on average, the largest difference among the tested conditions. Inset displays magnifications of the response traces within the time window highlighted by the shaded area. **(D)** Head lift translation velocity (upper trace, black) and rotation velocities during the flight shown in **(A)**. Velocity of rotation around the vertical head axis (yaw) in black, rotation around the frontal head axis (roll) in orange, and rotation around the transverse head axis (pitch) in green. The inset illustrates the axes of rotation and translation (same color coding as velocity traces; courtesy of C. Spalthoff).

We analyzed the responses of the wide-field motion-sensitive neuron V1, one of the fly's LPTCs, to naturalistic optic flow. The stimulus used in this study was generated from data obtained in a previous study (Hateren and Schilstra, 1999), in which the head position and gaze direction of a blowfly were monitored under semi-free flight conditions (see materials and methods). The flies were allowed to fly in a cubic box of 40 cm edge length with walls displaying photographs of herbs. The measured parameters allowed us to reconstruct what the fly had actually seen during flight. For the present study we chose flight trajectories that are known from a previous study (Karmeier et al., 2006) to cause strong excitation of the Vertical-System neurons VS1, VS2, and VS3, which are presynaptic to V1 (Kurtz et al., 2001). One of the fly's flight trajectories used for the present study is shown in Figure 1A. The head position (red dots) and head orientation are plotted every 100 ms. The reconstructed stimuli were replayed in a custom-built stimulus device, “FliMax” (Lindemann et al., 2003; Liang et al., 2011), which allowed us to present the image sequences in a panoramic way, with high maximum luminance (equivalent to midday outdoor brightness), and at high frame rates; thus, matching all the relevant criteria to mimic natural stimulation. All recordings were obtained from V1 neurons in the right brain hemisphere (termed “ipsilateral”). However, in addition to the recordings with the original version of the reconstructed image sequence we recorded a second set of responses, during which a mirror-inverted version of the visual input was presented (for simplicity briefly termed “contralateral” in the following). With this inverted input the right eye receives the input normally seen by the left eye and vice versa. This procedure allowed us to determine the responses of the V1 neurons in both brain hemispheres without the need to change the recording site.

### Impact of CDM and epinastine on neuronal responses to naturalistic optic flow

Several recent studies have concordantly shown that locomotor activity as well as systemic administration of octopamine or its agonist CDM elevates the resting activity of fly LPTCs and enhances their stimulus-induced responses (Longden and Krapp, 2009, 2010; Chiappe et al., 2010; Maimon et al., 2010; Jung et al., 2011; Haan et al., 2012; Rien et al., 2012; Suver et al., 2012). In some of these studies, the response boost was particularly strong at high temporal frequencies of grating motion (Chiappe et al., 2010; Longden and Krapp, 2010; Jung et al., 2011). Functionally, this state-dependent change was interpreted to adjust neuronal tuning to the range of high velocities encountered during locomotion (Longden and Krapp, 2011). To assess how neuronal responses under naturalistic, dynamic stimulus conditions are affected by this modulation of sensitivity we recorded the responses of V1 to the behaviorally generated stimuli before and after administration of CDM. Moreover, to verify that the observed changes are truly mediated by octopaminergic modulation, and to capture the full range of the octopamine-mediated sensitivity alteration, we additionally applied an antagonist of octopamine receptors, epinastine (Roeder et al., 1998). Epinastine was administered either directly after the untreated control recordings or following the recordings in the presence of CDM.

In Figure 1 we illustrate the modulatory effects of CDM and epinastine on V1's resting activity (Figure 1B) and on the responses induced by naturalistic stimulation (Figure 1C). As demonstrated before (Longden and Krapp, 2009, 2010; Jung et al., 2011; Rien et al., 2012), application of the octopamine receptor agonist CDM (10 μl of a solution containing 2.6 μM) leads to an increase in resting activity (resting activity before CDM application: 45.7 ± 16.78 spikes/s; after: 61.4 ± 12.59 spikes/s; *p* = 0.079; *N* = 14; Figure 1B, left). Subsequent administration of epinastine (10 μl of a solution containing 2 mM) reduced V1's resting activity significantly (after epinastine: 35.5 ± 11.94 spikes/s; *p* = 0.00001 compared to CDM application alone; *N* = 14; Figure 1B, left). When epinastine was applied directly after the untreated control recordings we also observed a decrease in resting activity (before epinastine: 48.1 ± 30.29 spikes/s; after: 32.6 ± 14.80 spikes/s; *p* = 0.08; *N* = 7; Figure 1B, right).

Figure 1C shows the mean responses averaged across all recorded V1 cells (*N* = 14) to a naturalistic optic flow sequence for the control condition and the different pharmacological treatments. The responses fluctuate irregularly between different levels of spiking activity, transiently reaching peak spike rates of more than 400 spikes/s, and only occasionally falling below the resting spike rate. Some of the prominent peaks of V1's spike rate are associated with rapid gaze shifts during abrupt changes in the fly's heading direction, called “saccadic turns” (Schilstra and Hateren, 1999). These saccadic turns manifest as strong deflections in the rotation velocities of the fly's head (Figure 1D, bottom) and occurred approximately every 100 ms under the given recording conditions (Kern et al., 2012). During the rest of the time (called “intersaccades”) the fly's movement is almost free of rotations and is therefore dominated by forward, sideward and lift translation. This saccadic flight and gaze strategy has been interpreted to facilitate the extraction of information from optic flow about self motion and about the spatial layout of the environment (Kern et al., 2005; Karmeier et al., 2006; Egelhaaf et al., 2012). Due to its sensitivity to vertical motion, the activity of V1 also depends on the lift velocity (Karmeier et al., 2003), which unlike the rotation velocities fluctuates in a fairly smooth way (Figure 1D, top). However, much of the fluctuation in V1's spike rate seems not to be obviously linked to any of the self-motion parameters alone, but rather to the current combination of parameters. Importantly, variability in the response is expected even at times with similar self-motion parameter constellations, because the fly's view of the arena is continually changing.

At course inspection, the response traces recorded before drug application (Figure 1C, black traces) as well as those recorded after application of CDM (red traces) and after subsequent epinastine application (blue traces) appear to reflect the flight trajectory in a qualitatively similar manner. Profound differences in the temporal profile of the responses are not evident, even when comparing the conditions with the largest amplitude differences, “CDM” (red traces) and “CDM + Epinastine” (blue traces). Consequently, most of the time the difference between these conditions (purple trace) remains much below 50 spikes/s, even for time intervals where V1's spike rate exceeds 300 spikes/s in one or both conditions. This increase by a factor of at most 1/6 contrasts with a more than threefold boost in response gain induced by CDM or locomotor activity obtained under certain stimulus conditions (Chiappe et al., 2010; Longden and Krapp, 2010; Jung et al., 2011; Rien et al., 2012). However, in these studies the sensitivity of LPTCs was assessed with constant-velocity grating motion, which was presented on displays that produce lower maximum luminance and cover a smaller part of the visual field than the “FliMax.”

Thus, as a first impression, octopaminergic modulation of the responses of V1 to naturalistic optic flow is weaker than expected from previous studies in which less complex visual stimuli were used. However, despite the overall similarity in the response properties between the investigated conditions, there are several subtle, but consistent, differences, which may be crucial for the functioning of the V1 cell in the context of the control of flight behavior. First, after CDM treatment the stimulus-evoked responses (Figure 1C, red traces) are most of the time above the spike frequencies in the control condition (black traces) and in the condition after subsequent epinastine application (blue traces; see also the corresponding difference traces shown in purple). Second, a close inspection of the time courses during abrupt upstrokes in spike rate indicates that these transients are slightly steeper and faster after CDM administration (Figure 1C, insets). This difference in response dynamics, which will be analyzed further below, is consistent with previous accounts on different LPTCs, showing that CDM reduced the neuron's latency at the onset of constant-velocity grating motion (Longden and Krapp, 2009, 2010; Haan et al., 2012).

### Octopaminergic modulation of spiking activity

**Figure 2:**
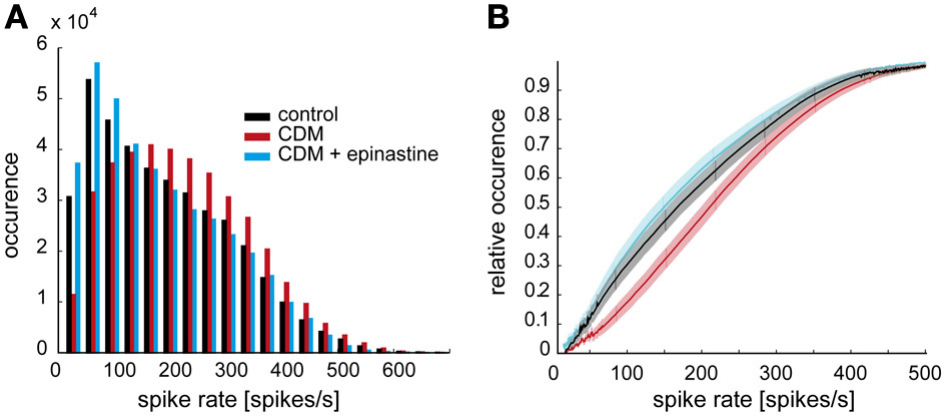
**Octopaminergic modulation of stimulus-induced overall spiking activity of V1. (A)** Frequency distribution of spike rates in response to naturalistic stimulation in the control condition (black), after CDM administration (red), and after subsequent epinastine administration (blue). **(B)** Empirical cumulative frequency distributions. The shaded areas around the lines indicate 5% confidence intervals. Same color coding as in **A**.

We analyzed how the overall activity of the V1 neuron during stimulation with naturalistic optic flow is affected by octopaminergic modulation. Following CDM application spike rates below 150 spikes/s occur less often, whereas the occurrence of spike rates higher than 150 spikes/s increases (Figure 2, black and red bars). Thus, the peak of the frequency distribution of the spike rates is shifted toward higher values by 50–100 spikes/s (Figure 2). To some extent, this change results from the fact that CDM treatment led to enhanced peak frequencies in response to saccadic flight manoeuvres (see Figure 1). The shift in the frequency distribution of the spike rates is reversed after subsequent epinastine application (Figure 2, blue columns). The modulation of spike activity by CDM and epinastine is also evident in cumulative frequency distributions of the spike rate (Figure 2B). Here, CDM treatment results into a rightward shift of the curve (cf. black and red lines), whereas after subsequent administration of epinastine (blue lines) the curve is positioned slightly left of the control curve. The statistical significance of these differences is evident from the fact, that the 5% confidence intervals (indicated by shaded areas around the curves) are clearly separated for large parts of the curves. Moreover, a sigmoid shape of the curve is visible for the “CDM” condition, but less pronounced for the other conditions. These changes in the shape of the distribution, which are also reflected in more symmetrical frequency histograms of the “CDM” condition compared to the other conditions (see Figure 2A), might be interpreted to indicate a better coverage of the entire available neuronal working range (van Hateren, 1997).

### Development of octopaminergic modulation in the course of naturalistic stimulation

**Figure 3:**
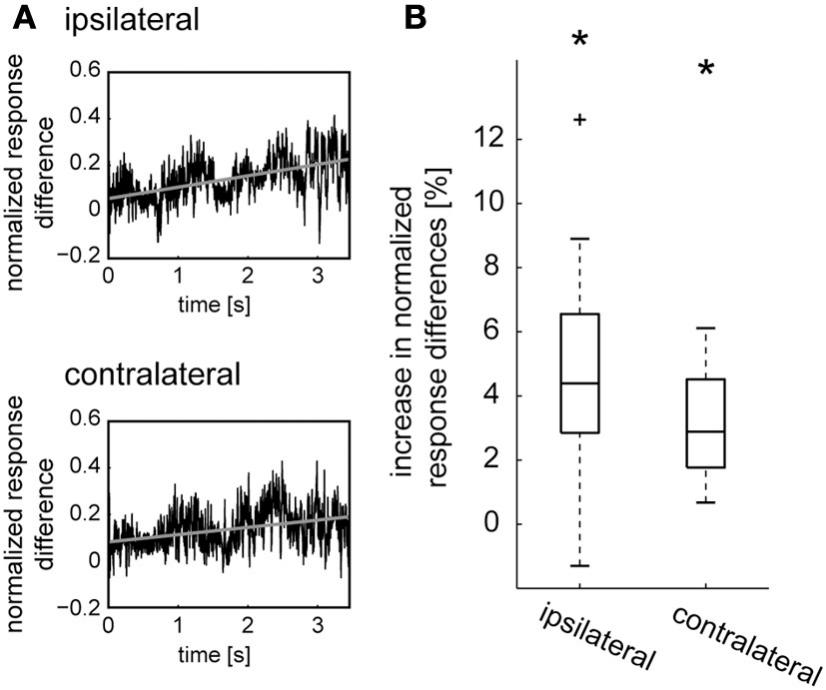
**Development of octopamine-mediated effects over time during sustained naturalistic stimulation. (A)** The difference between the responses of the V1 neuron in the states after CDM application and after subsequent epinastine application (see purple lines in Figure 1C) was taken and, for normalization, divided by the sum of the respective spike rates for each time point. The time courses of the normalized response differences obtained for each neuron were averaged and are shown for the ipsilateral and contralateral side (black traces). Linear regressions were calculated for each neuron and averaged (gray lines). **(B)** Percentage of increase per second in normalized response difference, determined from the slope of the linear regression line. The boxplots show the data of all cells, with the median as a central line, the upper/lower quartiles containing 50% of all data as box edges, and whiskers spanning the data range excluding outlier, which are shown as crosses. The asterisks represent statistical significance at the 5% significance level (Wilcoxon signed rank test, *N* = 14).

In previous studies CDM was shown to affect adaptation of LPTCs during sustained motion stimulation (Longden and Krapp, 2010; Haan et al., 2012; Rien et al., 2012). The effects of CDM and epinastine on the responses to naturalistic stimulation might therefore depend on stimulus history. Hence, we analyzed how these effects evolve in the course of stimulation. To obtain a relative measure of effect size we divided the time course of the difference in spike rate between “CDM” and “CDM + epinastine” by the time course of the sum of these spike rates. This normalization compensates to some extent for the strong fluctuations in spike rate, which would otherwise obscure small time-dependent changes of the magnitude of the pharmacological effect. In almost all recorded cells we observed a steady increase in the normalized response differences with ongoing stimulation. Figure 3A displays the average time course of the normalized response difference across all recordings. A linear regression analysis revealed a 4.4 ± 2.07% increase for the ipsilateral side (*p* = 0.0004) and a 2.9 ± 1.45% increase for the contralateral side (*p* = 0.00001; Figure 3B). This increase in the strength of octopaminergic effects with ongoing stimulation is consistent with a previous account, showing that CDM enhances the motion sensitivity of LPTCs by counteracting contrast gain adaptation (Rien et al., 2012). Effects that are due to the modification of adaptive properties are expected to depend on stimulation, and would therefore as seen in the present results, build up during sustained stimulation.

### Similarity between dynamic response profiles during different pharmacologically induced states

**Figure 4:**
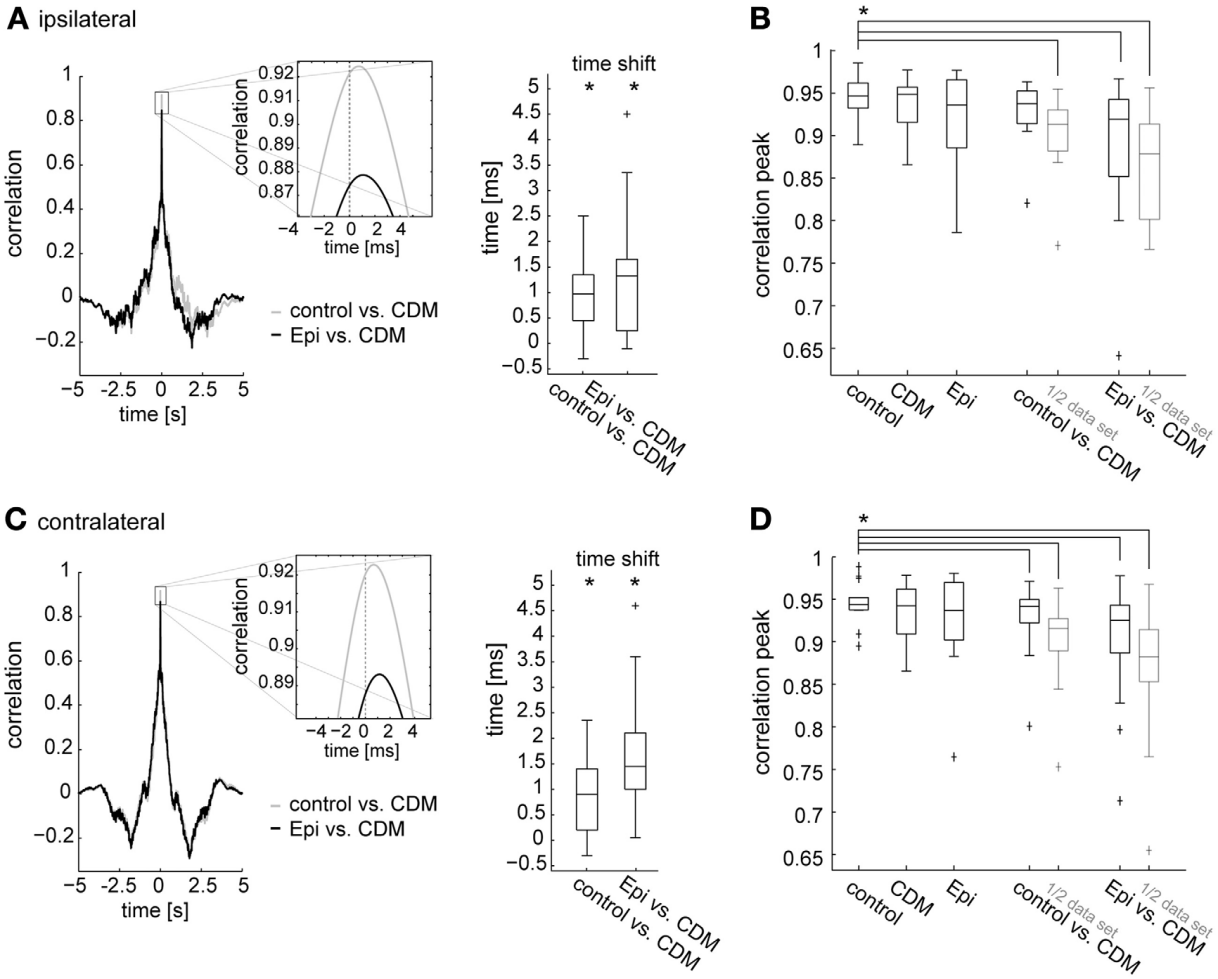
**Time-delayed cross-correlations calculated between different states of octopaminergic modulation and comparison with cross-correlations for responses recorded in the same state. (A)** Left, cross-correlation calculated between the responses of the control condition and following CDM application (gray), and between the responses after CDM and subsequent epinastine administration (black). Average across all flies for the ipsilateral V1 is shown. Inset shows a magnification of the correlation peak to illustrate the time shift. Right: time shift summarized for all recorded flies as determined from the cross-correlations. **(B)** Comparison of the maximum values reached by the peaks of the cross-correlograms calculated between the traces recorded in two different conditions (“control vs. CDM” and “Epi vs. CDM”) and between traces recorded in one and the same condition (“control,” “CDM” and “Epi”). For the correlation calculation within one condition, we divided each dataset into two parts of identical size and calculated the correlation. The same procedure was applied for the data denoted by “1/2 data set.” Note that, for conciseness, “Epi” is used to denote the condition in which epinastine was administered after application of CDM. **(C,D)** The same analysis for the contralateral V1 neuron. See legend of Figure 3 for an explanation of Box-Whisker plots. The asterisks represent statistical significance at the 5% significance level (Wilcoxon signed rank test, *N* = 14). In **(B)** and **(D)** significance is only indicated for the comparison of control with each other condition.

In the previous sections we showed that administration of CDM and epinastine induced systematic changes in spike rate during stimulation with naturalistic optic flow. Apart from these changes, which are also reflected in corresponding up- and downward shifts of the response traces (Figure 1C), the responses appear to be similar in their temporal profile across all conditions. To test this similarity quantitatively, and to test whether the CDM-induced decrease in latency observed during fast upstrokes in spike rate (Figure 1C insets) is effective throughout the response to naturalistic optic flow, we calculated time-delayed cross-correlations of the response traces. For each fly, the cross-correlations were calculated with average response traces for each treatment. Figure 4 shows the cross-correlograms between the responses recorded before and after CDM application (Figures 4A,C, left, gray traces), and between the responses recorded after CDM treatment and after subsequent epinastine application (Figures 4A,C, left, black traces). In all cases the correlograms reached high peak values above 0.9 for near-zero time shifts, indicating the close similarity between the temporal response profiles across all conditions. The correlation peaks were shifted from the zero line by about 1 ms, indicating slightly faster responses in the high-activity state mimicked by the pharmacological procedures. The shift of the peak in the cross-correlogram is 0.98 ± 0.48 ms (*p* = 0.001) when correlating the CDM with the control condition, and 1.33 ± 0.70 ms (*p* = 0.0006) when correlating the CDM with the epinastine condition (Figure 4A for the ipsilateral side; C for the contralateral side: w/o vs. CDM 0.9 ± 0.6 ms, *p* = 0.002; epinastine vs. CDM 1.45 ± 0.65 ms, *p* = 0.0001). The temporal positions of the peaks of the cross-correlograms are significantly different from zero across the cell sample (see boxplots in Figures 4A,C, right). Application of epinastine alone did not induce a significant shift in the cross-correlogram with the control condition (data not shown).

To validate that the temporal profiles of the responses to naturalistic optic flow remain fairly similar across the different states of octopaminergic modulation, we compared the maximum correlation values of the cross-correlations between the conditions with those of the cross-correlations calculated for the traces recorded within a condition (obtained by splitting the data sets into two halves). Notably, since average traces are formed from a limited number of responses which are inherently variable, the maxima reached in these within-condition cross-correlations remain below a value of “1” (Figures 4B,D, three leftmost columns). The maximum values of the between-condition cross-correlations (Figures 4B,D, two rightmost columns) are only slightly lower than those of the within-condition cross-correlations. In particular, we found a remarkably strong correlation between the responses recorded in the control condition and those recorded after CDM application (0.94 ± 0.016, ipsilateral; 0.94 ± 0.010, contralateral). A significant difference compared to the within-condition cross-correlation before drug application (“control”) was present only for the contralateral side (*p* = 0.008). Although the correlation between the response traces dropped after subsequent epinastine application it still remained well above 0.9 (0.92 ± 0.041, *p* = 0.0002; ipsilateral; 0.92 ± 0.035, *p* = 0.002 contralateral). When calculated with halved data sets, the peak cross-correlation values between the conditions dropped consistently by a small amount and were all significantly lower than the within-condition values before drug application (control vs. CDM 0.91 ± 0.021, *p* = 0.0001; CDM vs. epinastine 0.88 ± 0.041, *p* = 0.0001, ipsilateral; control vs. CDM 0.92 ± 0.014, *p* = 0.0001; CDM vs. epinastine 0.88 ± 0.031, *p* = 0.0002, contralateral).

**Figure 5:**
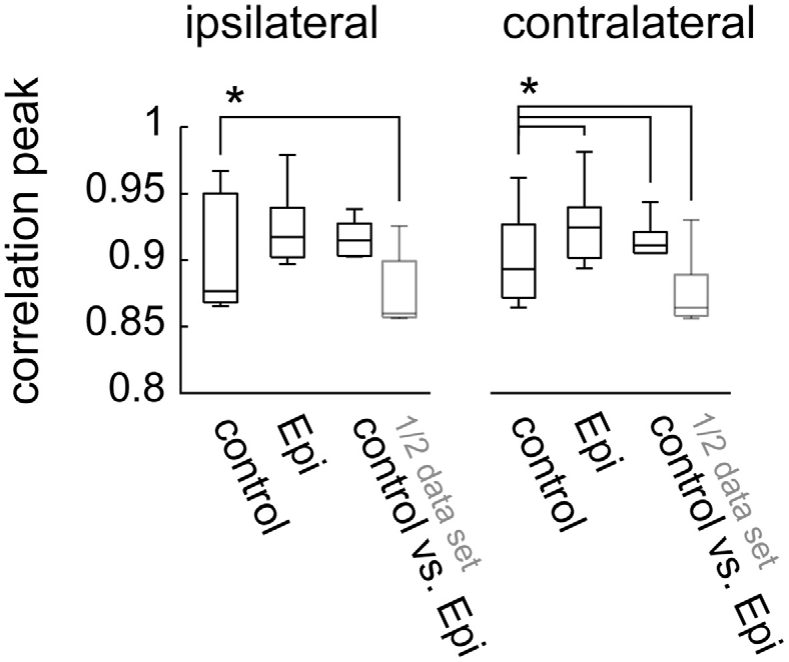
**Peak cross-correlation values for the visual responses within and between conditions for experiments (*N* = 7) in which epinastine was applied alone, without previous application of CDM.** Data presentation as in Figures 4B,D. The asterisks represent statistical significance at the 5% level (Wilcoxon signed rank test, N = 7)

In a smaller number of flies (*N* = 7) epinastine was administered alone, i.e., without beforehand applying CDM. In line with the previous results, the maximum cross-correlation values between the responses before and after drug application reached values close to 0.9 (Figure 5). Only for the halved data sets these values were significantly lower (*p* = 0.016) than the control values (based on halved data set of the responses before drug application).

**Figure 6:**
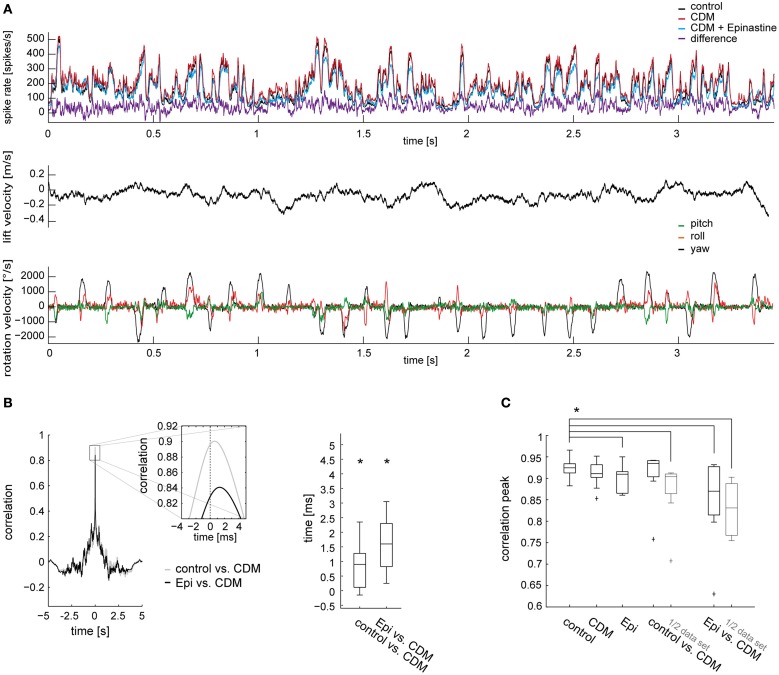
**Similarity of neuronal responses in different states of octopaminergic modulation evaluated with another naturalistic optic flow sequence. (A)** Averaged responses of ipsilateral V1 neurons (*N* = 9) before drug application, after CDM application, and after subsequent epinastine application (top) and self-motion parameters of the fly's head (middle and bottom). Data presentation and color coding as in Figure 1. **(B)** Time-delayed cross-correlations calculated between different states of octopaminergic modulation and comparison with cross-correlations for responses recorded in the same state. Left, cross-correlation calculated between the responses of the control condition and following CDM application (gray), and between the responses after CDM and subsequent epinastine administration (black). Average across all flies for the ipsilateral V1 is shown. Inset shows a magnification of the correlation peak to illustrate the time shift. Right: time shift summarized for all recorded flies as determined from the cross-correlations. **(C)** Comparison of the maximum values reached by the peaks of the cross-correlograms. See legend of Figure 4 for explanations. The asterisks represent statistical significance at the 5% significance level (Wilcoxon signed rank test, *N* = 9).

To corroborate that the findings outlined in the preceding sections are valid across different optic flow sequences we performed the same analysis as described above with data obtained from another flight trajectory. We obtained basically the same results as with the first flight sequence. This was the case for the ipsilateral (Figure 6) as well as for the contralateral V1 neuron (data not shown). Similar to the first flight sequence, the cross-correlation between the response traces in the different states (Figure 6B) peaked at values close to 0.9 (Figure 6C). As for the first flight sequence, the peak of the correlation was slightly shifted from the zero line, indicating faster responses in the high-activity state (0.9 ± 0.45 ms, *p* = 0.02, when correlating the CDM with the control condition, and 1.6 ± 0.75 ms, *p* = 0.0039, when correlating the CDM with the epinastine condition).

### Linear vs. non-linear nature of octopaminergic effects assessed by coherence analysis

**Figure 7:**
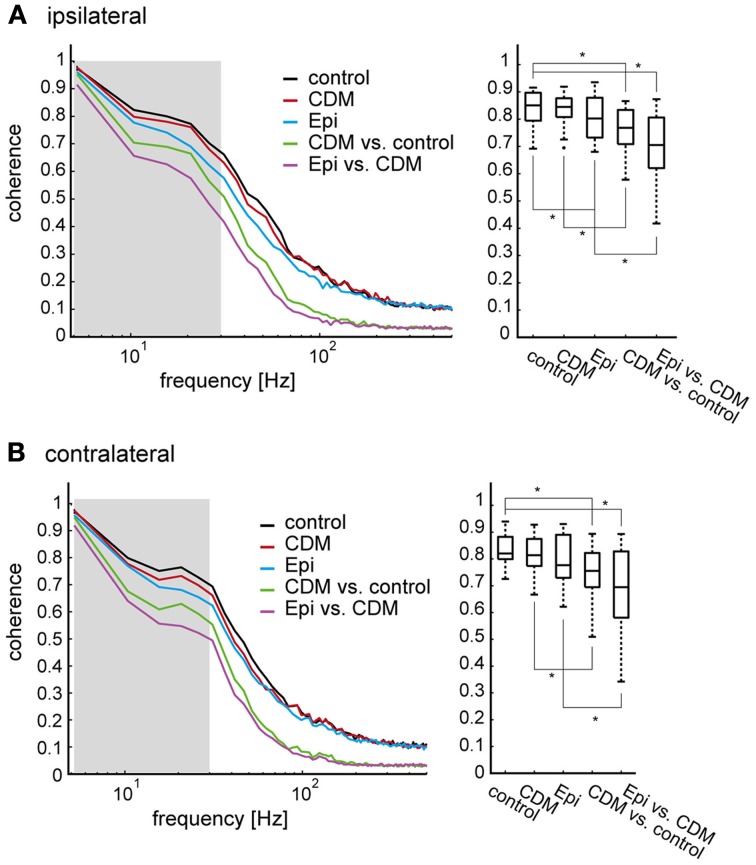
**Coherence of responses within and between different states of octopaminergic modulation. (A)** left: The coherence as a measure of noise (expected coherence) was determined as the coherence between individual responses and their mean response, all within one and the same condition. All coherence values for the ipsilateral side were first calculated for individual flies, then averaged across all flies (*N* = 14). The black trace shows the control condition, the red trace the condition after CDM administration, and the blue trace the condition after subsequent epinastine administration. To determine the combined effect of noise and non-linearities induced by the pharmacological manipulation the coherence was determined between the mean response of the control condition and each individual recording after CDM application (green), and between the mean response following CDM application and each individual recording after subsequent epinastine application (purple). Right: Coherence values averaged in an interval from 0.5 to 30 Hz, as indicated by the shaded area in **(A)**. See legend of Figure 3 for an explanation of Box-Whisker plots. Note that, for conciseness, “Epi” is used to denote the condition in which epinastine was administered after application of CDM. **(B)** The same analysis for the contralateral side. The asterisks represent statistical significance at the 5% significance level (Wilcoxon signed rank test, *N* = 14).

We performed a coherence analysis to further characterize how the dynamic response characteristics of the V1 neuron are affected by CDM and epinastine. The coherence quantifies how well two time series, for example two neuronal response traces, can be transformed into each other by linear filtering operations. As the coherence is a function of frequency, the quality of such a transformation can be determined for the different frequency components of the time series. In a perfectly linear and noise-free system the coherence equals “1” for all frequencies. To characterize the effect of CDM we calculated the coherence between the individual response traces following CDM application and the average response trace in the untreated control condition (Figures 7A,B, left, green traces; averaged across flies, *N* = 14). The coherence is close to “1” for low frequencies, declines gradually to a value of about 0.7 at 20 Hz and then starts to drop steeply at higher frequencies. Slightly lower values throughout the entire frequency range are obtained when the coherence is calculated between the individual response traces obtained after subsequent epinastine administration and the average response trace of the CDM-treated condition (purple traces).

A coherence value below “1” can either result from non-linearities in the relationship between two time series or from the presence of noise. To estimate how much of the deviation of the coherence values from “1” is not linked to an effect of the pharmacological manipulation, but can rather be attributed to noise in the responses to the naturalistic stimuli, we determined the so-called expected coherence. The expected coherence represents the coherence between individual response traces and the corresponding averaged response. Thus, an expected coherence below “1” indicates system noise. In Figure 7 the expected coherences, averaged across flies, are shown for the untreated control (black traces) and after drug application (red and blue traces for CDM and subsequent epinastine application, respectively). Although the expected coherences are consistently slightly larger than the coherences between conditions, they still remain much below “1” over most of the frequency range. As the expected coherences represent the impact of noise they provide an upper bound for our evaluation of how strongly the response dynamics of V1 are affected by CDM and epinastine in a non-linear way. Although such effects are present, they appear to affect the response variability across different pharmacological conditions a lot less than trial-to-trial variability (i.e., noise) even within a condition.

For a systematic comparison we averaged the coherences in the low frequency range (0.5–30 Hz, as indicated by the shaded areas in Figures 7A,B, left). Within the expected coherences the lowest values were found after epinastine administration (Figures 7A,B, right). The low expected coherence after epinastine application indicates an increase in noise, which is likely to result from the overall decrease in spike rate (Kalb et al., 2008). However, comparing the mean coherence values with the expected coherences forming the respective upper bounds reveals significant drug effects, which cannot be attributed to an increase in noise alone. For the ipsilateral side a mean coherence between the untreated control and the CDM condition of 0.71 ± 0.055 compares to an expected coherence for the CDM condition of 0.80 ± 0.034 (*p* = 0.0001; corresponding values for the contralateral side: 0.69 ± 0.076 vs. 0.76 ± 0.069, *p* = 0.0023). A similar trend exists when comparing the mean coherence between the CDM and the epinastine condition with the expected coherence for the epinastine condition (ipsilateral: 0.65 ± 0.106 vs. 0.75 ± 0.073, *p* = 0.00001; contralateral: 0.63 ± 0.124 vs. 0.72 ± 0.097, *p* = 0.00001). It is important to note that the limited size of the data sets induces a systematic bias. Expected coherence values tend to be overestimated, because the average response and the single response traces stem from the same data set. In contrast, different data sets form the basis for the coherence calculation between different conditions. Thus, the coherence values and the expected coherence values might actually be even more similar than estimated by our analysis.

In summary, the coherence analysis shows that octopaminergic modulation leads to non-linear changes in the responses of V1 during naturalistic stimulation. However, as indicated by the finding that expected coherences are much lower than “1,” the similarity between individual response trials appears to be more severely limited by neuronal trial-to-trial variability (i.e., noise) than by octopaminergic modulation.

### Types of self-motion responses affected by octopamine

**Figure 8:**
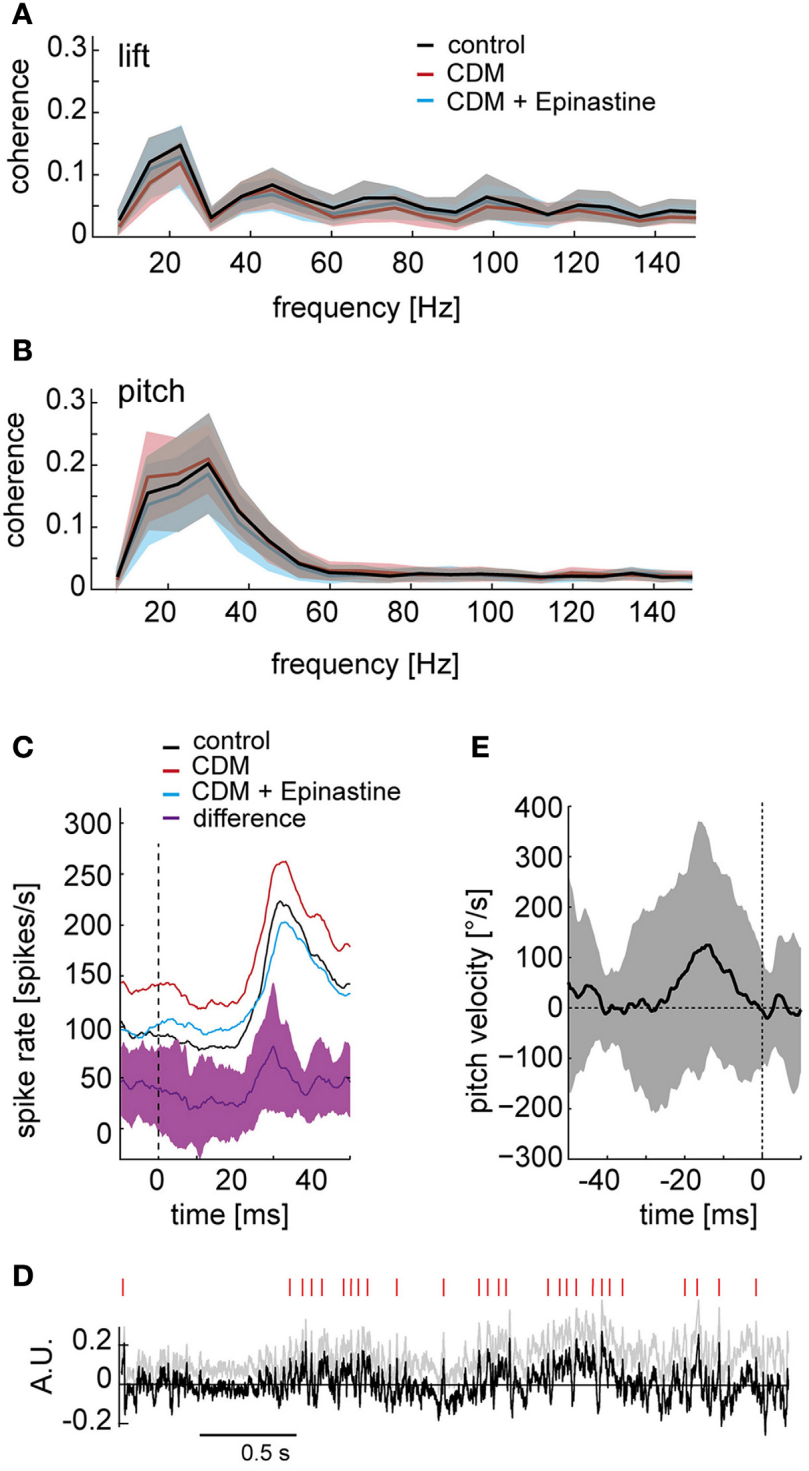
**Octopaminergic modulation of responses to particular self-motion parameters. (A)** Coherences between the lift translation in intersaccadic intervals and the neuronal responses. Control condition depicted in black, after CDM application in red, and after subsequent epinastine application in blue. The shaded areas indicate the standard deviations. **(B)** The same analysis for pitch rotation. **(C)** Responses to high velocities around the pitch rotation axis (pitch saccades) shown for the contralateral V1 averaged across all recordings and flies. The black line shows the average response of the control recordings, red line after CDM application, blue line after subsequent epinastine application. The purple line indicates the average difference between the responses after CDM application and following subsequent epinastine application. The shaded area depicts the standard deviation of the response difference. The vertical dashed line marks the time point of the pitch saccade's peak velocity. **(D)** Detection of points in time, during which the difference between the responses after CDM application and after subsequent epinastine application reaches a certain threshold. For an unbiased detection of threshold crossings, the positive slope of the normalized difference trace (gray, same data as in Figure 3A) was corrected by subtracting the regression line (see gray lines in Figure 3A), resulting in the trace shown in black. Red dashes on top indicate when the signal crosses a threshold of 1.7 times the standard deviation. **(E)** Average pitch velocity around the time points of threshold crossings of the difference signal (marked by the vertical dashed line at time “0”). Standard deviation indicated by shaded area. All data for the contralateral side.

Being part of the vertical system, the V1 neuron is most sensitive to downward motion in the frontal visual field and is therefore thought to be involved in sensing up- and downward translation of the fly as well as rotation around the pitch axis (Krapp et al., 2001; Karmeier et al., 2003). We therefore asked whether the representation of particular self-motion parameters by the neuronal responses is affected by octopaminergic modulation. To this aim, we calculated the coherences between the neuronal responses and the self-motion parameters. In general, these coherences were fairly low, because the response of V1 depends on several self-motion parameters, rather than only on a single one, as well as on the properties of textures in its receptive field (see also Karmeier et al., 2006). Exemplarily, the values obtained for pitch and lift, are shown in Figures 8A,B, respectively, (only data for contralateral side shown). The coherences were almost identical across the different pharmacological conditions. For the ipsilateral side, the overall coherence values were even lower, and indications for differences between conditions were not present. The same was true for the coherence of the neuronal response with other self-motion parameters (data not shown). Thus, our analysis suggests that the relationship between the self-motion parameters and the neuronal response is not markedly affected by octopaminergic modulation.

Above we have shown that the overall coherence between distinct self-motion parameters and the responses of the V1 neuron is similar across the different pharmacological conditions. Nevertheless, it might be possible that more pronounced differences can be discerned when the analysis is focused on distinct events of the fly's locomotor pattern. Hence, we scrutinized whether the response transients (see Figure 1C, insets) caused by fast rotations around the transverse head axis (“pitch saccades”) are subject to octopaminergic modulation. Figure 8C shows the averaged response triggered by the peak of pitch saccades over all stimulus repetitions and flies. Only for the contralateral side, but not for the ipsilateral side (data not shown), the peak in pitch velocity is followed by a brisk rise in spike rate after a latency of about 30 ms. The difference between ipsi- and contralateral saccade-triggered averages may result from differences in the visual input to the left and right eye when flying close to the arena walls (see Figure 1B). Throughout the pitch-saccade-triggered average response CDM administration leads to an upwards shift of the trace by 40–50 spikes/s (Figure 8C, cf. black and red line). In contrast, after subsequent epinastine administration (blue trace) the modulation depth of the response is reduced. These differences become particularly strong during the rising edge of the response transient, as seen most clearly in the difference trace between the response after CDM application and the response after subsequent epinastine application (Figure 8C, purple trace). The size of this difference does not just vary with the response strength, but peaks during the upward deflection in spike rate and falls back to a lower level well before the maximum in spike rate is reached. This finding suggests that, if a pitch saccade is associated with a sharp response transient, the slope of its upstroke may be amplified by octopaminergic modulation.

We additionally used a reverse approach to search for types of self-motion, during which the response of the V1 neuron is markedly affected by octopamine. To this aim, we took the normalized response differences (as shown in Figure 3), and detected the time points at which these differences exceeded a threshold level (1.7 times standard deviation). For this analysis, we compensated the overall slope of these difference traces based on a linear regression (see gray lines in Figure 3A) to avoid an increase in the number of threshold crossings with stimulation time. We then assessed the average self-motion velocities in a 50 ms time window starting 40 ms before the detected peaks in the normalized, slope-corrected difference traces (Figure 8D; peaks are indicated by vertical red lines). Figure 8E shows the average rotation velocity around the pitch axis in this time window for the contralateral side. In spite of high variability, an increase in pitch velocity seems to precede the detected peaks in the difference trace by approximately 20–30 ms, which is in accordance with the effect of octopamine on the pitch-saccade-triggered responses (Figure 8C). A similar, but weaker correspondence was found for the ipsilateral side (data not shown). Average rotations around the other axes of rotation and average translations were not obviously related to the peaks in the difference trace (data not shown).

## Discussion

There is growing evidence that an animal's behavioral state strongly influences the neuronal response gain and dynamics of visual information processing (review: Maimon, 2011). However, how the processing of the optic flow perceived under a natural condition is affected by state-dependent modulation is still unresolved, because in all previous experiments periodic gratings drifting with experimenter-designed velocity profiles were used to address state-dependent modulation. These stimulus conditions contrast with flight in a structured environment, which confronts the fly with highly complex optic flow, determined by its flight movements, its gaze shifts, and the textures in the surroundings. At the same time, there is convincing evidence that the state dependence of visual motion processing in dipteran flies is mediated by the neuromodulator octopamine (Longden and Krapp, 2009; Jung et al., 2011; Suver et al., 2012). In the present study we investigated octopaminergic modulation of spiking activity of the large-field motion-sensitive V1 neuron of the fly during naturalistic visual stimulation. Whereas previous studies aimed to clarify the mechanisms underlying locomotion-induced and octopaminergic modulation of neuronal optic flow processing (Maimon et al., 2010; Jung et al., 2011; Haan et al., 2012; Rien et al., 2012; Suver et al., 2012), we focus on its functional relevance. We were able to address this issue by reconstructing what a fly has actually seen during semi-free flight and presenting these stimuli during neuronal recording. This approach preserves the flight-induced dynamics of optic flow, which is shaped by the typical alternation between sustained intervals of translation and brief periods of high rotational velocities (saccades).

### Octopaminergic modulation of responses to naturalistic optic flow compared to simpler motion stimuli

As judged by the cross-correlations and coherences between the responses in different pharmacologically induced states (Figures 4–7) the temporal response profiles of the V1 neuron during naturalistic stimulation are not extensively altered by state-dependent modulation. This result is important for the interpretation of previous conclusions based on experiments in which naturalistic optic flow was used without considering the fly's activity state (Kern et al., 2005; van Hateren et al., 2005; Karmeier et al., 2006; Liang et al., 2012). Based on the results obtained in the present study, extensive re-evaluation of the conclusions drawn in the former studies about the use of optic flow for self-motion analysis as well as for the extraction of information about the structure of the environment seems unnecessary. On a cautionary note, one must however, acknowledge that further experiments are needed to clarify whether the results obtained in the present study for the V1 neuron can be transferred to other LPTCs.

In previous studies a strong boost of neuronal gain was observed in the aroused state, in particular when analysing the steady-state response to a grating drifting at a high velocity (Chiappe et al., 2010; Longden and Krapp, 2010; Jung et al., 2011). In contrast, our results indicate that the state-dependence of the responses to naturalistic optic flow appears to be less pronounced. A potentially lower efficiency of octopaminergic modulation induced by systemic drug administration compared to genuine locomotor activity is for several reasons unlikely to account for this difference. First, similarly strong effects were observed when directly comparing both procedures in the same set up (Jung et al., 2011; Suver et al., 2012). Second, using exactly the same pharmacological procedure as in the present study we observed strong changes in the gain of the V1 neuron when responding to constant-velocity grating motion (Rien et al., 2012). Third, we made the attempt to capture the full range of octopaminergic modulation by using the specific octopamine antagonist epinastine.

Taken together, the most likely explanation for the fairly weak octopaminergic modulation during naturalistic stimulation lies in the stimulus properties themselves. Using a panoramic display with ultrabright LEDs the brightness and the contrast of the presented patterns come close to those observed under midday outdoor conditions. Luminance contrast is a relevant parameter because previous studies have shown that the contrast gain of fly LPTCs is affected by the octopamine agonist CDM (Haan et al., 2012; Rien et al., 2012). Contrast sensitivity of fly LPTCs is decreased after adaptation with motion of a high-contrast grating. Although both studies on octopaminergic modulation of contrast gain adaptation (Haan et al., 2012; Rien et al., 2012) showed that this adaptation component is modulated by octopamine, they differed with respect to the type of effect. We found that CDM counteracts contrast gain adaptation of the neurons H1 and V1 in *Calliphora*, thus, leading to an increase in response gain (Rien et al., 2012). In contrast, Haan et al. (2012) reported that contrast gain adaptation in LPTCs of hoverflies (*Eristalis spec*.) was stronger after administration of CDM. As this result is hard to reconcile with the boost in response gain caused by octopamine in *Calliphora* and *Drosophila*, it remains open whether octopamine exerts different functions in *Eristalis*. Assuming that CDM in the present study reduced contrast gain adaptation, the presence of high-contrast textures during most of the presentation time of the naturalistic stimulus might explain why the consequence of this change of adaptive properties is less pronounced than during presentation of test gratings with often much lower contrast (Haan et al., 2012; Rien et al., 2012).

Stimulus-specific differences in the strength of adaptation, which may be relevant in the context of octopaminergic modulation, were also demonstrated in a previous study (Kurtz et al., 2009). In this study, the H1 neuron was driven into a strongly adapted state by sustained grating motion with constant properties and then challenged by sudden changes in one of the stimulus properties (velocity, direction, luminance contrast, or pattern wavelength). These stimulus discontinuities still induced strong response transients even when the neuron's overall spiking activity was reduced much by adaptation. Sudden changes of stimulus properties are present almost all the time in the naturalistic stimulus used in the present study. Therefore, adaptation-induced reductions of neuronal activity can be expected to be weaker for a naturalistic stimulus than for constant-velocity gratings. The same would then be the case for octopaminergic modulation, given that it acts on neuronal adaptation.

### Octopaminergic modulation of response dynamics to naturalistic optic flow

A controversial aspect of the state-dependence of visual motion processing is whether the velocity tuning of fly LPTCs shifts toward higher values during locomotor activity. Whereas in some studies the boost in response gain was larger at high than at low temporal frequencies of grating motion (Chiappe et al., 2010; Jung et al., 2011), the changes of response gain were rather uniform across a large range of temporal frequencies in other studies (Longden and Krapp, 2010; Suver et al., 2012). As discussed in Rien et al. (2012) these discrepancies might be explained when considering the dependence of contrast gain adaptation on temporal frequency and the different analyses time windows evaluated in the different studies. It is difficult to transfer these findings to naturalistic stimulation, because in contrast to drifting gratings contrast frequencies are not fixed for irregular image sequences, and even the velocity is never uniform across the visual field. Nevertheless, one might expect that strong changes of the velocity tuning by octopamine would result in pronounced changes of the time course of the neuronal response and, thus, reduce the correlation between the responses recorded during different states. We indeed found that some of the cross-correlation and coherence values between different states were significantly lower than control values characterizing the response variability within a state. However, the similarity of single response trials is already strongly affected by within-state variability, as seen in the large difference of the expected coherences from “1” (see Figure 7). Compared to this, the additional effects caused by octopaminergic modulation appear to be fairly weak. Altogether, our findings suggest that octopaminergic modulation of the dynamic properties of the V1 neuron might be more prominent during sustained motion stimulation than under the unstable stimulus conditions of free flight.

In the present study, we found a small but consistent decrease in response latency after administration of CDM, which could be fully reversed by subsequent application of epinastine (see Figure 4). Similar effects were previously demonstrated for the responses of the blowfly neurons V1 and V2 to a dot moving on a circular path (Longden and Krapp, 2009) and for the responses of several fly LPTCs at the onset of grating motion (for blowfly V1 and V2 see Longden and Krapp, 2009; for blowfly H2 see Longden and Krapp, 2010; for hoverfly HS see Haan et al., 2012). Depending on the stimulation protocol, type of LPTC and fly species the decrease in latency is in the range of 1–4 ms, and thus, smaller than the decrease obtained for *Calliphora* H1 neurons upon raising the ambient temperature by about 10°C (Warzecha et al., 1999). Nevertheless, the decrease in latency might be functionally beneficial to support fast motor responses to optic flow cues and to limit instabilities during closed-loop optomotor regulation (Warzecha and Egelhaaf, 1996). A further benefit might result from the modulation of overall spike rates by octopamine. The more pronounced sigmoid shape of the cumulative frequency distribution of the spike rates after CDM application compared to the untreated condition (see Figure 2) suggests that octopaminergic modulation results in a better coverage of V1's entire working range. This shift in the frequency distribution of spike rates might be of functional relevance to efficiently exploit the neuron's information capacity in the high-activity state, but saving energy in the low-activity state.

### Conflict of interest statement

The authors declare that the research was conducted in the absence of any commercial or financial relationships that could be construed as a potential conflict of interest.
